# Supplementary material for: Are carbon emissions trading and green financial instruments synergistic? -Comprehensive quantitative research based on content analysis
Source: PLoS One. 2024 Mar 7;19(3):e0298601. doi: 10.1371/journal.pone.0298601 (PMC10919601; doi:10.1371/journal.pone.0298601)
Supplement: S1 Data — (ZIP) [file pone.0298601.s001.zip › Data/地级市Z_F工作报告.zip!/╔╜╬≈/╜·╓╨╩╨/└┤╫╘╣1⁄2╓┌║┼í╛┬φ┐╦╩2╛▌═°í┐_2012.docx]

各位代表：

　　现在，我代表市人民政府，向大会报告政府工作，请各位代表审议，并请各位政协委员提出意见。

　　一、2011年工作回顾

　　2011年，我市坚持以科学发展为主题，以转变经济发展方式为主线，以建设全省“四化”率先发展区为统领，按照“速度快、效果好、特色浓、人民富”的总体要求，奋力拼搏，扎实工作，圆满完成了全年各项目标任务，实现了“十二五”良好开局。

　　——经济实力进一步增强。在国内经济保持较快增长、我市经济连续两年高位运行的基础上，8项主要经济指标保持两位数增长，4项指标增速20%以上，4项指标增速高于全国、全省平均水平。全市地区生产总值890.2亿元，人均达到4227美元；规模以上工业增加值431.8亿元，财政总收入178.1亿元，一般预算收入78.0亿元，全社会固定资产投资580.2亿元，社会消费品零售总额325.4亿元，外贸进出口总额2.3亿美元，城乡居民人均收入分别达到18918.6元、6912.5元。上述指标全部超额完成省定考核目标。

　　——发展质量进一步提高。单位生产总值能耗预计下降3.7%，6种主要污染物减排完成年度目标，所有县（区市）环境空气质量全部达到国家二级标准，市城区综合污染指数1.45、下降4.61%，林木绿化率提高1.2个百分点，全部超额完成省定任务。全市新认定高新技术企业3家、累计达到14家，授权专利337件，增长4%。全市工业经济效益综合指数216.6,同比提高33.5个百分点，创历史新高。

　　——居民生活继续得到改善。农民人均纯收入增幅创近十年来最高，超过城镇居民收入增速，城乡收入差距由2010年的2.82：1缩小为2.74：1。转移农村劳动力6.79万人，同比增长61.7%。居民消费价格总水平上涨4.7%，分别低于全国、全省平均水平0.7个、0.5个百分点。城乡居民储蓄存款达到933.3亿元，比上年增长16.2%。

　　——县域经济发展更加协调。6个县（区市）财政总收入超10亿元，灵石率先突破30亿元。东山地区生产总值、固定资产投资、财政总收入3项指标增速分别高于平川2.1个、20.6个、12.2个百分点，区域发展更加协调。

　　各位代表，我向大会郑重报告，市三届人大二次会议确定的各项目标任务圆满完成，市政府向全市人民承诺的实事全部兑现！

　　一年来，我们主要做了以下工作：

　　项目建设取得新突破。全面落实项目储备、签约、落地、建设推进措施，新增储备项目投资额2200亿元、累计达到1.45万亿元。深入开展大招商、大引资活动，引进新项目197个，资金到位491亿元、到位率全省第一。全市542项重点工程，完成投资590.6亿元，同比增长29.5％。第二、第三产业投资分别比上年增长32.2%、34.8%，在全部投资中所占比重分别提高0.8个、1.4个百分点；在第二产业中，非煤产业投资增长37.4%，新兴产业投资增长102.4 %，非国有投资增长37.7%。扎实开展工业项目推进年活动，全市500万元以上工业项目投产127项。项目建设有力地拉动了经济增长，带动了投资优化，推动了产业升级，为长远发展打下了坚实基础。

　　转型发展迈出新步伐。坚持以煤为基、多元发展，晋中特色现代产业体系建设呈现出许多新亮点。资源型产业巩固提升，煤炭资源整合煤矿兼并重组步伐更加扎实，全年地方煤矿生产原煤5074.6万吨，创历史最高；焦化行业整合重组启动；新增电力装机容量230万千瓦，全省第一；冶金行业素质提升，安泰120万吨H型钢、太谷恒达高碳铬铁投产达效；煤层气开采利用、太阳能发电、风力发电等新能源产业初露头角；甲醇、尿素等一批煤化工项目及电石项目进展顺利。非煤产业加速壮大，装备制造、食品、新材料、高新技术等产业取得新进展。七大示范工业园实现销售收入增长26.9%。现代服务业增添新活力，太铁货运项目获批，方略保税物流中心功能延伸工程获准，全市物流业增加值占GDP比重13.5%，比上年增加0.1个百分点。文化旅游发展出现新气象，大型实景演艺印象团队落地平遥，首开与北方地区合作先河，国家旅游服务业标准化示范城市创建扎实推进，旅游总收入增长19%；《褒尚论道》等一批文化精品力作获得国家级大奖，国家文化生态保护区“大晋中”文化旅游核心区等重大规划启动，全市文化产业增加值占到GDP的4.85%，保持全省领先水平。深入开展品牌和标准化建设，6个企业和单位获评首届“市长质量奖”及提名奖，新增“宏艺”、“榆缆”、“永隆”三个中国驰名商标；纺织机械和玻璃器皿两个国家级产品质量检验中心获准筹建。房地产业投资78.9亿元，比上年增长17%。全市第三产业增加值完成328.2亿元，人均超过1万元，步入快速上升通道。

　　“三农”工作取得新进展。全市财政涉农支出达到46.6亿元，占一般预算支出的30.2%，其中市本级财政安排“三农”资金4.4亿元，为历年最多。耕地得到有效保护，粮食总产达15.8亿公斤，创历史新高，蔬菜、肉蛋奶总产继续位居全省第一。晋中现代农业示范区30个年度重点项目全部开工，16个省级项目全部完工，完成投资10亿元。主要粮食作物综合机械化水平达到65.6%，比全省高10个百分点。全市建成“一村一品”专业村273个、专业乡镇15个，新发展设施蔬菜6.14万亩。太谷通宝进入国家级龙头企业行列。新增农村专业合作社869个，率先在全省实现全覆盖。开展农民增收明白卡工作的乡镇新增43个，覆盖率达到45.8%，指导农民增收的作用进一步发挥。扎实开展领导干部下乡住村活动，又有1.5万贫困人口脱贫。集中连片打造新农村建设示范区20个，建设中心村30个。新增23个卫生镇、225个卫生村，80%的乡镇、15%的村创建成市级卫生乡镇、卫生村。寿阳、灵石成为全省县域经济发展先进县。

　　城乡面貌有了新变化。大力推进市域城镇化进程，太原晋中同城化发展加速，城镇化率提高1.62个百分点、达到45.74％。突出规划引领，5大类80个重大规划项目编制完成，北部新城、太榆科技创新城、山西高校新校区、现代物流园区、山西新能源汽车·机械装备制造区、潇河生态景区规划加快实施。扩容提质步伐加快，全市铺开各类市政重点工程193项，其中市城区完成投资61.6亿元，是上年的7.8倍，超过“十一五”投资总和；市城区启动国家园林城市创建工作，介休市通过国家园林城市初步验收，潇河生态公园等40项重点园林项目进展顺利，人均公园绿地新增1.05平方米、达到10.89平方米，城市品位和承载力不断强化。全市市容环境卫生进一步改善，11个县（区市）再次全部通过省级卫生城市验收。基础承载能力强化，“双百”水利工程全面铺开，山西大水网东山供水工程在全省率先上马，石膏山水库、恋思水库建设加快推进，松塔水电站大坝封顶下闸蓄水，昔阳松溪供水工程奠基开工。汾邢、阳黎高速晋中段全速推进，龙城高速通车在即。电网建设、“气化晋中”稳步推进。精心打造“双百公里林果带、千里林业生态圈”，完成造林合格面积35万亩。

　　各项改革打开新局面。综改试验工作全面启动，完成市县两级综改方案，“108综合发展廊带”规划进一步推动，灵石成为全省首批综改试验试点县，介休、灵石成为全省首批扩权强县试点县，介休义安镇成为全省唯一的全国经济发达镇行政管理体制改革试点，10项重大转型标杆项目得到确立。一批重点领域的改革逐步深化，争取到城乡建设用地增减挂钩、矿业存量建设用地整合和露采矿山企业用地三项改革政策，新增用地指标上万亩。扎实推进全省“资本市场改革创新示范区”建设，小贷组织推广、村镇银行设立、农村信用联社转制农村商业银行、中小企业天交所上市、私募股权投资基金设立等七项工作全省第一。林权制度、文化体制、国有企业、基层医疗卫生机构等改革取得突破。

　　民生社会事业实现新发展。财政用于民生支出占全市一般预算支出的比重达到70%，总量比上年增长34.9%。农村新的“五个全覆盖”工程7项任务中，5项提前一年实现全覆盖、2项完成省定年度任务。城镇新增就业4.44万人，年末城镇登记失业率1.78%。开始实施“社会保障一卡通”，使民生社会保障有了便捷途径。城镇社会保险综合覆盖率93%，新型农村养老保险参保率99.1%，新农合参合率97.86%，农民看病难、看病贵问题进一步得到缓解。城乡低保实现应保尽保，低收入群体享受价格性补贴6046万元。“爱心煤”政策得到落实，74.1万户农民家庭受益。晋中市和左权县分别再获“全国双拥模范城”称号。中小学校舍安全工程全部完工，新改扩建标准化公办幼儿园60所，义务教育均衡发展继续保持全国领先。全民体育健身蓬勃开展，女子长拳获得晋中体育史上首个世界冠军。全市开工保障性安居工程3.31万套，主体完工1.23万套，完成投资29.51亿元，全部超额完成省定目标。加强安全生产风险预防管理，安全生产形势平稳好转，煤炭生产百万吨死亡率、亿元GDP生产安全事故死亡率分别下降21%、14.3%。创新食品安全监管机制建设，切实加强食品链条全过程风险预防控制、全过程监督问责，食品安全责任监督工作试点经验得到国务院领导充分肯定。国防建设和其它各项社会事业都有新进步。

　　民主法制和精神文明建设得到新加强。市政府自觉接受市人大及其常委会的法律监督、工作监督和市政协的民主监督，共办理206件人大代表建议、339件政协委员提案。打响创优发展环境“新晋中战役”，出台优化政务环境、提高行政效能的12条规定，简化政府投资项目审批管理，大力推进投资建设项目阶段式联合审批，市内审批时限大幅缩短。“六五”普法全面启动。进一步加强政府自身建设，完善重大决策规则和程序，审计和监察工作得到强化，廉政建设取得新成效。第九届村委会换届顺利完成。深入开展群众性精神文明创建活动。进一步加强和创新社会管理，扎实开展安全监管、社会管理、居民自治网格化建设和知责、履责、问责教育，着力排查化解各类社会矛盾，依法打击违法犯罪活动，治安案件同比下降9.9%，社会保持和谐稳定。

　　各位代表，一年来，我们集中谋划了一批管长远的大事，出台《加快建设全省“四化”率先发展区的决定》，编制完成“十二五”规划纲要、晋中市城市总体规划等重大规划，进一步理清了“十二五”发展思路。集中开展了一批利当前的实事，坚持经济运行分析调度、每月安全生产例会制度，多次集中观摩项目建设，组织千名干部赴太原、孝义考察，进一步推动思想大解放，重点工作督查实现常态化，创新开展重点区域领域纠风治乱集中整治活动，大打优化发展环境攻坚战，保障了经济运行健康有序。集中推进了一批增后劲的要事，举全市之力加快推进山西高校新校区、晋商公园等一批同城发展标杆项目；大力建设龙湖立交桥、龙城高速、太谷旅游机场等一批重大基础设施标杆项目；引进落地吉利新能源汽车、中航特种装备及车辆、太重榆液高性能液压产品基地、太钢万邦镍铬合金、中科鸿基生物科技、平遥煤化新型光学材料等一批重大产业转型标杆项目，进一步夯实了转型跨越的基础。一年内获得国家级荣誉称号、国家级工程17个，提升了晋中在全国的知名度和影响力。

　　各位代表，取得的成绩来之不易。这是中共晋中市委统揽全局、正确领导的结果，是市人大、市政协大力支持、有效监督的结果，是全市人民辛勤工作、共同努力的结果。我代表市人民政府，向全市人民，向驻地部队广大指战员、武警官兵和国家、省驻地单位，向各民主党派、工商联、各人民团体和社会各界人士，向所有关心和支持晋中发展的同志们、朋友们，表示衷心感谢和崇高敬意！

　　在肯定成绩的同时，我们也清醒地认识到：我市产业结构偏重、新兴产业不足，多元发展格局尚未形成；支撑转型跨越发展的标杆项目不多、推进不快的问题仍然存在；节能减排压力加大，保持城乡居民收入持续较快增长难度增加，改善民生、创新社会管理和加强安全生产监管的任务仍很艰巨；部分干部思想和作风不适应转型跨越、率先发展要求等。我们必须采取更加有力的措施认真加以解决。

　　二、2012年主要工作

　　今年是实施“十二五”规划承上启下的重要一年，是全面贯彻落实我市第三次党代会精神的关键之年，是党的十八大胜利召开之年，做好今年工作意义重大。当前，尽管国际经济形势严峻复杂，国内经济增长下行压力和物价上涨压力并行，我市经济持续向好的基础尚不稳固，做好经济工作的难度加大，但我们仍处于中部崛起、综改试验、同城发展、转型跨越、“四化”率先等多重机遇叠加期，加快发展的机遇大于挑战。我们必须切实做到头脑更加清醒、谋划更加周密、准备更加充分，抢抓机遇、迎难而上，稳增长中快发展，快发展中调结构，奋力推动晋中转型跨越、率先发展迈出新步伐、取得新成就！

　　今年政府工作的总体要求是：全面贯彻落实省第十次党代会和市第三次党代会精神，以科学发展为主题，以加快转型跨越为主线，以建设全省“四化”率先发展区为统领，按照转型发展先行区、晋商文化复兴地、现代宜居生态市的发展定位，落实速度快、效果好、特色浓、人民富的总要求，紧抓综改区建设和太原晋中同城化发展机遇，把稳中求进、更好更快发展作为首要任务，着力在项目建设、产业转型、同城推进、文化繁荣、综改试验、民生改善、社会建设等方面取得新进展，以优异成绩迎接党的十八大胜利召开。

　　主要预期目标是：地区生产总值增长13%；规模以上工业增加值增长17%；全社会固定资产投资增长26%；财政总收入增长17%，一般预算收入增长12%；外贸进出口总额增长5%；社会消费品零售总额增长17%；城镇居民人均可支配收入增长10%；农民人均纯收入增长14%。这些是指导性指标，可以根据形势变化适当调整。

　　约束性指标是：居民消费价格涨幅控制在4%左右，城镇登记失业率控制在4.2%以内，万元GDP能耗下降3.5%，万元GDP二氧化碳排放量下降3.7%，二氧化硫、化学需氧量、氨氮排放量分别下降2%、1.3%、1%，氮氧化物排放量与上年持平，烟尘、粉尘排放量均下降3%。这些是硬任务、硬指标，必须完成。

　　实现上述目标，今年要重点抓好十个方面工作：

　　（一）强力推进项目建设。

　　毫不动摇地坚持抓项目就是抓发展的理念，坚持以项目建设总揽经济工作全局，坚持政策聚焦、要素聚集、力量聚合，落实“项目落地年”要求，强化重点项目储备、签约、落地、建设推进机制，以项目建设支撑转型跨越发展。

　　做大项目规模。着力策划、储备、引进、落地、建设一批标杆项目、标杆工程，率先突破、引领示范。今年安排省市重点项目511项，总投资3807.6亿元，年度计划投资710亿元以上，重点推进“工业新型化百强项目”，带动工业完成投资400亿元。平遥煤化新型光学材料、太重榆液高性能液压产品基地项目确保6月份部分投产，中科鸿基生物科技项目9月份试生产，吉利汽车确保年底完成冲压车间土建封顶、涂装车间设备安装、10月份焊接与总装车间土建开工，加快东方希望铝业、太钢万邦镍铬合金项目建设进度，力争中航特种装备车辆项目上半年开工建设。

　　扩大招商引资。瞄准招大商、引大资目标，坚持三个“三分之一”工作机制，落实招商奖励办法，着力将各级领导干部推向招商引资主战场，夯实以政府为主导、企业为主体、专业队伍为骨干、中介组织为辅助、园区为载体的招商机制。科学谋划一批100亿元以上的重大转型项目，拓展和创新招商途径，坚持“走出去”、“请进来”，以企招商、以商招商、项目招商、组团招商、会展招商，重点搞好与中央企业、知名民营企业对接，着力引进国内外战略投资者，加强与国际友好城市(地区)的交流合作,确保实现1500亿元招商引资目标，确保资金到位495亿元。注重招才引智，以项目为媒介，打包引进专业人才、先进理念和管理模式。

　　强化要素保障。创新供地机制，扎实推进城乡建设用地增减挂钩、矿业存量建设用地整合、露采矿山企业用地和集体土地先征后转“四项改革”，确保8个土地增减挂钩试点县4500亩周转指标落实到位，重点盘活挖潜闲置低效利用土地，全面实施“2065”农村土地整治工程。破解环境容量制约，优化总量分配机制，推行节能量、排污权、碳排放权有偿使用和交易，为项目建设提供保障。

　　加快项目建设。全面实行项目化管理，严格“5W1H”工作方法，落实项目法人、招标投标、工程监理、合同管理、政府监督等各项机制。严格实施领导包项目责任制，总投资10亿元以上项目由市级领导挂帅包扶，深入开展以百项定责、百组督查为主要内容的推进项目创优环境“双百行动”。加大项目建设各环节流程业务培训，落实投资建设项目阶段式联合审批办法，分类优化项目备案、审批、核准工作流程，强化跟踪监督和管理考核，确保按预测目标缩短审批时限。

　　各位代表,大上项目、上大项目、上好项目是实现转型跨越、率先发展的根本保证和坚实基础。我们必须举全市之力、行非常之策，聚万众之心、尽非常之责，在招商引资、招才引智、加快项目建设上取得新突破！

　　（二）加快经济结构转型升级。

　　着力改造资源型产业。煤炭产业要巩固扩大重组整合成果，继续抓好标准化矿井和企业团队建设，全年地方煤矿生产原煤5500万吨以上，百万吨死亡率控制在0.2以下，完成基建技改投资100亿元以上，稳定全市经济基本面。焦化产业要大力推进兼并重组，以介休、灵石为主，全市规划建设6大焦化循环经济园区，优化发展6个热、气源焦化企业，扶持壮大8大焦化兼并主体和单独保留企业，焦炭产能保持在2500万吨左右。电力产业要加快华能左权电厂二期、榆次瑞光二期以及秸秆发电、风力发电、瓦斯发电等新能源项目前期工作，运用价格政策推动转型发展。

　　倾力发展非煤产业。加快发展具有比较优势、带动能力强的装备制造、新材料、高新技术、食品加工和现代物流等转型产业，努力提高新兴产业占比率。装备制造业要支持经纬纺机整合重组区域内各类纺机资源做强做精，推进高行、方盛等一批自主创新能力强的液压企业集聚，大力发展精密铸造和高端液压元件，全力加快华成柳工、百成电动车、晋东煤机等项目建设。食品加工业要扩大今麦郎饮品、双合成食品等企业市场份额，确保雪花啤酒、燕京啤酒应有份额，加快福源昌老陈醋“醋博园”建设，实现醋产业规模突破。新材料产业要重点推进山西陆源10万吨金属镁及镁铝制品等重大项目建设。高新技术产业要启动晋中科技创新产业园规划，推进左权宏远科技新建1万吨聚酰胺制品等项目实施。现代物流产业要以推进太铁货运中心、方略保税物流中心功能延伸工程等7个重大项目为重点，加快三晋综合保税物流港成型发展。加强房地产价格调控，促进房地产市场健康发展。

　　致力提升文化旅游产业。设立文化产业发展专项资金，重点支持旅游开发、产业园区、特色工艺品、精品剧目、演艺表演等8大类46个重点项目建设，推动文化产业增加值占GDP比重再提高0.2个百分点。加大旅游营销投入，加强建设项目管理，《印象平遥》确保按时首演，办好第十二届平遥国际摄影大展，加快平遥推光漆文化产业创意园、祁县艺术玻璃文化产业园建设，推进平遥古城、乔家大院、绵山5A级景区创建，推进榆社云竹湖生态文化旅游开发，全年旅游总收入达到150亿元以上，成为转型发展的新亮点、新支撑。

　　大力发展产业集聚区。加快产业集聚区规划建设，提高产业集聚区支撑发展、引领转型、要素集聚和项目支撑的能力。要完成国家级晋中经济技术开发区规划编制，申报省级大寨经济开发区。加快山西新能源汽车·机械装备制造区水电路等基础建设，积极引进布局新的项目，太重榆液要进一步发挥对全市液压产业的龙头作用，每个县工业相对集中区都要入驻新项目。落实园区科学发展行动方案，建立以投资强度、投入产出率、土地收益、就业和税收贡献等指标为主体的考评体系，切实发挥园区经济的带动作用。大力发展循环经济，不断延伸煤焦化、煤气化、煤电铝、煤电材产业链条，资源就地转化率、原材料深加工率和传统产业循环率今年都要有新提高。

　　全力扩大内需外贸。积极发展居家养老、社区照料、月嫂早教等家庭服务业，推进再生资源回收利用体系建设，完善鲜活农产品流通体系，提升“万村千乡”市场工程、“新网工程”和“农超对接”，用足“家电下乡”政策。稳步拓展对外贸易，抓好祁县玻璃器皿全国外贸转型升级示范基地建设，扩大轻工、机电、陶瓷、农产品等优势商品的出口份额，拓展东盟、俄罗斯、拉美等新兴市场，培育外贸竞争新优势。

　　努力推进“大中小微”企业协同发展。建立经济运行分析联席会议制度，千方百计保障煤电油气运以及土地、资金、人力等生产要素的供应，确保经济平稳健康运行。支持大企业做强做优，新增销售收入过50亿元企业1户，销售收入过20亿元企业达到9户、过10亿元企业达到22户，铸造产业集群销售收入达到60亿元、纺机集群达到50亿元、新能源汽车（专用车）集群达到30亿元、液压件和玻璃器皿集群分别达到20亿元。实施中小企业成长工程，出台促进中小企业成长扶持办法，建立市县两级中小企业发展专项资金，清理整顿涉企收费，推进公共服务平台、政银企担保合作平台和创业基地建设，全年新孵化培育小微企业500户，新增销售收入过亿元“小巨人”民营企业5—10个，新培育销售收入3000万元到5000万元骨干企业20户，扶持中小微企业健壮成长。

　　各位代表，结构调整永无止境，转型发展任重道远。只要我们坚持以煤为基、多元发展，全力构建具有晋中特色的现代产业体系，就一定会在转型跨越的大路上越走越快、越走越好！

　　（三）毫不放松抓好“三农”工作。

　　进一步加大强农惠农富农政策支持力度。今年市本级财政投入“三农”资金5.2亿元，重点扶持设施蔬菜等特色现代农业发展；不折不扣落实省出台的农业灌溉水价补贴等“五项补贴政策”、设施蔬菜大县奖补等“八项惠农政策”、规模健康养殖等“七项强农工程”和高标准农田建设、农机推广普及等“双十强农惠农工程”。切实加强农田水利建设，新增改善灌溉面积10万亩。严格耕地保护制度，做好土地整理开发工作，改造中低产田和盐碱地，建设、新造、复垦土地12万亩，提高粮食和农业综合生产能力。

　　进一步发展特色现代农业。扎实推进晋中现代农业示范区建设，突出抓好12个重点项目建设，完成投资10亿元以上，重点提升太谷国家现代农业示范县、榆次国家农业科技园区的示范引导力。扩大“一村一品、一县一业”规模，新建200个专业村、5个专业乡镇、52个特色万亩基地，着力打造20个工厂化养殖企业和200个标准化养殖园区，新发展干果经济林10万亩、设施蔬菜6万亩，打造榆次、太谷两个10万亩设施蔬菜基地县。抓好榆次丰润泽、太谷巨鑫等16个科技示范园项目，培训农民100万人次，农业科技贡献率、农机综合机械化水平分别提高2个、3个百分点。落实国务院扶持农产品加工龙头企业政策，重点跟踪服务9个省级特色现代农业战略支撑项目，抓好20个千万元以上农产品加工改扩建项目和榆次汇隆、太谷金谷2个农产品批发市场建设，全市农产品加工销售收入突破100亿元。建设100个旗舰型合作社联合体，加快形成“龙头企业+基地+中介组织+农户+标准化”、“龙头企业+商标（地理标志）+农户”等产业化经营格局，提高市场竞争力。

　　进一步推进新农村建设。全面完成农村新“五个全覆盖”任务，继续开展创“三优”活动，建设“六美”乡村100个。全市推广普及农民增收明白卡。进一步加大扶贫开发力度，落实新十年扶贫纲要，坚持开发式扶贫方针，实施板块推进战略，突出抓好左权、榆社、和顺三县片区开发项目，完成易地扶贫搬迁5300人。深化领导干部包村增收活动，帮助群众解决实际困难。大力实施区翼发展战略，加强县域基础设施建设，布局一批强县富民的好项目、大项目，加快县域社会事业发展，壮大县域综合实力。

　　各位代表，“三农”工作是政府全部工作的重中之重。我们一定要怀着对农民群众的深厚感情，瞄准“十二五”收入翻番、消除人均纯收入5000元以下县的目标，打牢基础，增加投入，提高收入，竭尽全力让农民群众过上幸福美好的新生活！

　　（四）实质性推进同城化建设。落实省委、省政府要求，加快城镇扩容提质，全面提升市域城镇化水平。

　　强化规划管理。重点推进山西高校新校区综合服务配套和核心景观、太榆科技创新城战略研究等6大类68项规划项目。理顺“两级政府、三级管理、四级网络”的城管体制，加快城市管理信息平台与社会管理网格平台对接，推进数字化城管进程。

　　加快同城对接。落实“一个规划统筹、一个标准建设”要求，主动与太原融合对接，健全工作沟通和协调机制，积极开展太原晋中共建区规划研究，引进战略合作伙伴，力争太榆科技创新城项目启动建设；统筹推进两市供水、供气、污水处理、金融、通讯等基础设施建设和服务一体化进程，重点加快汇通路北段拓宽改造工程，力争开工第二污水处理厂二期工程。加强榆次区、太谷县、祁县、寿阳县与太原相邻县区对接协同。

　　提升市城区。继续实施“北进、西联、南扩、东延”扩城计划，全力推进市城区7大类76项市政重点工程，今年完成投资165亿元。全力加快山西高校新校区两横五纵道路及地下管网设施配套建设，确保9所高校今年9月有一个年级以上学生入驻的需要，年底完成规划建筑总面积80%以上的建设任务。加快实施城市规划展示馆、汽车客运总站、汇通大厦、民俗风情商业街等北部新城项目建设；完成东出口、顺城街地下空间综合利用等工程；加快城中村改造，推进直隶庄、大东关、北关片区改造和小南庄、侯坊村整体搬迁。

　　打造大县城。按照扩大规模、增强实力、完善功能、形成特色的原则，继续完善县城市政基础设施，确保污水处理厂稳定运行，加快垃圾处理厂、城市星级公厕和集中供热项目建设运行，进一步增强城镇承载能力，提高县城辐射带动作用。

　　建设特色镇。继续抓好义安、胡村两个省级示范镇建设，全面加快建制镇供水、供气、供热、环卫等基础设施建设。认真落实省户籍制度改革意见，着力破除农民融入城市的体制机制障碍。

　　夯实基础设施支撑。引深“双百”水利工程建设，加快实施东山供水工程，完成和顺恋思、灵石石膏山、左权泽城西安3处应急水源建设，推进昔阳松溪供水工程，抓好引黄入市和南扩等项目前期。加速完善市域交通网络，龙城高速、汾邢高速平遥至左权段今年7月竣工通车，年底贯通阳黎高速晋中段，推进318省道董坪沟至郜家庄50公里一级路改建工程、汾屯线香乐至左家堡10公里二级公路改建工程，完成农村公路改造150公里。加大电网改造力度，新规划晋中经济技术开发区220千伏变电站、新开工榆次北500千伏等输变电工程，新增35千伏及以上输电线路540公里、变电容量386.6万千伏安。

　　各位代表，太原晋中同城化是我们独特的现实机遇，投资晋中就是投资太原，我们一定要以太原晋中共建区和太榆科技创新城为抓手，高标准规划、高起点建设、大力度实施，为实质性推进太原晋中同城化奠定坚实的基础！

　　（五）加强生态环境保护。坚持节能、减排、增绿、保洁四措并举，构建蓝天、碧水、绿地生态系统，加快城乡生态化步伐，增强可持续发展能力。

　　全方位推进节能降耗。继续开展能效对标，推行合同能源管理、节能低碳行动计划等节能新机制，抓好节能技术项目建设，严格执行固定资产投资项目节能评估制度，深入推进各领域节能工作。继续依法淘汰高能耗、高污染行业落后生产能力，重点抓好不达标水泥企业关停淘汰工作。加强节能执法工作，做好资源综合利用、清洁生产和循环经济增长，推动低碳经济发展。

　　严要求推进治污减排。围绕年内建成省级环保模范城目标，全面实施污染减排、污染防治“双百工程”。积极推动污水深度治理、脱硫脱硝和防尘抑尘改造；加强市城区和108国道沿线炉窑集中整治，加快垃圾无害化处理、污水处理厂提标升级改造和中水回用工程建设，抓好农村环境连片整治试点示范，强化环境执法监管，严厉打击违法排污行为，努力改善城乡环境质量。

　　大力度推进造林绿化。以创建省级林业生态市为抓手，搞好城乡绿化、厂矿区生态修复治理、碳汇造林等工程，全年完成造林合格面积37万亩。扩大城市园林绿化规模，加快市城区晋商公园三期、潇河公园二期、社火公园和植物园等项目建设，引深县城和重点集镇园林绿化创建活动，加快祁县昌源河国家湿地公园建设，完成介休国家级园林城市和寿阳、昔阳省级园林城市创建。

　　进一步引深城乡环境卫生整治行动。推进环境卫生管理城乡一体化，加大环卫投入力度，按照生活垃圾“村收集、乡转运、县处理”的环卫运行体制，开展清洁示范县、示范乡镇、示范街村和卫生标兵乡镇、标兵村创建活动，力争灵石县、左权县率先通过国家卫生县城验收。加快“气化晋中”建设步伐，完成东山过境煤层气管线扫尾工程，11个县（区市）城区实现煤层气、天然气、焦炉煤气气源管网覆盖，启动汽车加气站网络工程建设。

　　各位代表，拥有良好的生态环境，是全市人民的共同期盼。我们必须对子孙后代和未来负责，竭尽全力让晋中大地天更蓝、地更绿、水更清、空气更清新、环境更宜人！

　　（六）持续保障改善民生。

　　坚持教育优先。全面实施《晋中教育规划纲要》，加大教育投入力度，深化国家级义务教育均衡发展改革试点工作，加快推动市域教育由初步均衡向基本均衡、优质均衡跨越。积极发展职业教育和成人教育，支持高等院校发展。抓好国家级教育信息化试点项目，加快实施中小学“班班通”和数字化校园建设。大力推进中小学标准化建设，年内至少有一半以上县（区市）通过省政府新一轮标准化验收。

　　促进充分就业。实施更加积极的就业政策，全面做好各类人员的就业工作。市级和寿阳、太谷要建成省级创业型城市，昔阳、平遥要分别建成国家级、省级农村劳动力转移示范县。大力实施创业培训，扶持更多有创业意愿和创业能力的人自主创业，以创业带动就业。优化公共就业服务平台，积极开展就业援助和服务活动，动态消除零就业家庭。

　　提高保障水平。全面落实社保扩面和补助提标政策，提高统筹层次和保障水平，全市试行五险“一票征收”。继续加强住房公积金的扩面征缴，积极争取国家项目贷款试点。严格执行被征地农民预存款制度，规范农村低保救助程序。着力稳定物价总水平，进一步完善社会救助和保障标准与物价上涨挂钩联动机制，切实保障低收入群体的基本生活。加大保障性安居工程建设力度，落实项目法人终身责任制，以“阳光”租售为重点，完善保障房建设、分配、管理和退出机制。

　　发展各项社会事业。实施文化惠民工程，推进市级群众艺术馆免费开放，确保县级文化馆、图书馆正常开放，完成613个20户以下已通电自然村广播电视村村通工程，深入开展送戏、送电影下乡活动，大力促进艺术精品生产，加快市级图书馆、博物馆建设，健全和完善公共文化服务体系。大力推进品牌文化战略，组织实施八大文化节庆活动，加快“大晋中”文化旅游核心区和晋中文化生态保护实验区规划实施。继续稳健推进医改工作，新农合参合率达到98%以上，完善基本药物制度，运行基层医疗机构新机制，加快市中医院和寿阳、灵石县人民医院试点改革进程。坚持深化食品安全责任监督机制，加大学校食品安全监管力度，提高食品药品安全保障水平。落实全民健身计划，搞好“体育进农村”活动，筹建中国太极柔力球国际交流平台。进一步做好人口和计划生育工作，稳定低生育水平，提高人口素质。大力发展福利和慈善事业，加快残疾人社会保障体系和服务体系建设。落实党的民族宗教政策，做好外事、侨务、对台等工作。支持国防和军队建设，健全完善军地联合指挥体系，发展人民防空事业，做好双拥和优抚安置工作。加强妇女儿童、老龄和红十字会等工作。

　　各位代表！保障和改善民生是我们义不容辞的责任，努力做到像抓经济建设一样抓民生保障，像落实经济指标一样办好民生实事，加快推进速度，加大落实力度，确保民生持续改善！

　　（七）抓好安全生产工作。

　　强化安全理念。牢固树立预防为主理念，坚持“每日从零开始”，把“生命至上、安全第一”要求，落实到建设、生产、经营、管理的全过程和各个环节。不折不扣落实国务院和省、市一系列安全生产制度，坚持每月安全生产例会和风险排查治理，完善安全生产的领导格局、监管体制、责任体系，巩固分工明确、责任到人、齐抓共管的工作局面。

　　深化打非治违。以煤矿、非煤矿山、尾矿库、危险化学品、民用爆炸物品、道路交通、特种设备、建筑施工、消防、烟花爆竹、冶金、电力为重点，在全行业、全领域严厉打击非法生产经营建设行为，坚决治理纠正违规违章行为，坚决杜绝重特大事故，有效遏制较大事故，努力减少一般事故。

　　落实主体责任。严格落实政府安全监管和企业安全生产主体责任，进一步完善安全生产挂牌责任制，做到职责分工明确、监管不留死角、责任落实到人。切实加强企业安全标准化建设，严格安全生产准入，做到安全设施与主体工程同时设计、同时施工、同时投产使用，打牢本质安全基础。加大投入保障力度，积极推进安全乡村创建，健全乡村安全监管体系。扎实开展安全生产知责、履责、问责教育轮训和“双万人”培训，夯实安全生产基层基础工作。落实安全生产网格化责任，借鉴食品安全责任监督工作试点经验，推动实施企业安全风险管理，加强生产经营的过程控制，形成风险排查治理长效机制，确保安全风险可控、在控。

　　严格奖惩考核。把安全生产作为考核各级各部门和企业的重要内容，严格实行“一票否决制”。实施过程问责，严格事故责任追究，保证各项安全生产措施落到实处。

　　各位代表，安全生产是事关转型跨越、率先发展的大事，也是最大的民生！我们只有认真履责、恪尽职守、勤勉工作，才能够实现安全生产形势的明显好转、稳定好转、根本好转！

　　（八）加快推进综改试验区建设。抓住“先行先试”这个最大政策，大胆探索实践，拓宽发展领域，创新体制机制。

　　搞好转型综改试点。全面实施2012年转型综改行动方案，强化国家级、省级开发区和“一市两县”、“一市两园”、“一县一企”先行试点的示范带动，加快22个转型综改标杆项目建设，扎实推进产业转型、生态治理、城乡统筹、民生社会四大任务和项目审批、用地保障、资金筹措等综合配套改革，加快“108综合发展廊带”规划建设，打造全省转型综改集中试验区，介休要争取列入全省绿色综改试验区，率先推行碳汇交易和排污交易制度。

　　加快金融创新。围绕全省资本市场改革创新示范区建设，积极推进中小企业股权融资、上市融资，争取天津股权交易市场在我市设立山西分市场；加快建设新型投融资平台，扩大金融机构信贷支持，稳步推进地方金融机构改革发展，支持银行业务创新，强化金融服务实体经济的能力；吸引资本市场各类中介机构落户我市；发展私募股权基金，推动民营资本向产业资本转化；培养本土的上市做市商、保荐人队伍，引导更多民间资本进入资本市场；积极引导煤焦领域退出的闲置资金投向实体经济，就地进行再投资、再创业；综合运用各种融资工具，采取BOT、BT等模式，吸引更多的资本投入转型跨越发展。

　　强化科技人才支撑。继续实施项目引导、载体建设、资金扶持和服务支撑四大科技工程，重点支持引导纺机、液压等产业提升，推进纺织机械和玻璃器皿两个国家级产品质量监督检验中心建设，加强企业研发能力，新培育2家高新技术企业，新建5个农业科技研发中心，建成1个省级国际科技合作基地。加大创新创业人才和急需紧缺人才的培养力度，鼓励校企合作，实施订单式人才培养，大胆引进一批高端人才和拔尖人才，努力营造人才辈出、人尽其才、才尽其用的创业环境。

　　深化各项改革。灵石、介休要深入推进扩权强县试点改革，用好用足市级下放的9大类85项管理权限，切实增强自主发展的能力和活力。重点推进市属建设集团、金恒公司、药材系统和市直部门企业改革，鼓励和引导民营企业积极参加国有企业的改制重组，建立市本级国有资本经营预算体系，切实加强国有资本收益管理。继续引深文化体制改革，加大政府对国有文化企业的扶持力度，制定和落实经营性文化单位改制后相关配套政策，引导、鼓励、支持民营经济、社会资本积极参与文化产业发展，大力培育合格的文化市场主体。积极稳妥推进事业单位分类改革，全面完成分类工作。深化集体林权制度改革，全面完成林权发证任务，完善公益林补偿等配套政策。

　　各位代表，敢字当头、先行先试是率先发展的强力引擎。我们一定要抓住综改试验这个最大政策，解放思想，深化改革，扩大开放，为全市转型跨越发展提供强劲的源动力！

　　（九）积极推进民主法制和精神文明建设。

　　发展社会主义民主政治。认真执行人大及其常委会的决议、决定，依法向人大及其常委会报告工作，自觉接受人大法律监督、工作监督和政协民主监督，认真办理人大代表建议和政协委员提案。支持各民主党派、工商联、无党派人士以及工会、共青团、妇联等开展工作。完善基层民主管理制度，提升基层自治组织的管理能力和水平。

　　加强社会主义法制和精神文明建设。加强和改进政府法制工作，支持人民法院、检察院依法独立公正行使职权，维护社会公平正义。加强法制宣传教育，进一步扩大法律援助知晓率、受援面，做到应援尽援，深入推进“六五”普法工作，在全社会形成自觉学法尊法守法用法的良好氛围。广泛开展群众性精神文明创建活动，积极推进移风易俗，进一步加强社会公德、职业道德、家庭美德和个人品德教育，营造文明健康、积极向上的社会风气。

　　全力维护社会稳定和公共安全。进一步加强和创新社会管理。以5大类43项社会管理创新项目建设为抓手，全力构建安全监管、社会管理、居民自治网格化建设和知责、履责、问责教育社会管理工作体系，提升组织指导、责任监管、事件处置能力，打造责任网、连心网、服务网。严格落实信访责任制，完善矛盾纠纷排查调解机制，畅通群众利益诉求渠道，解决群众反映强烈的热点难点问题。深入推进“平安晋中”建设，完善社会治安防控体系，严厉打击各类违法犯罪行为，搞好流动人口、特殊人群的管理服务，最大限度提高人民群众的安全感和幸福指数，维护社会和谐稳定。

　　（十）进一步加强政府自身建设。

　　发展的重任、人民的期待，对政府工作提出了更新更高的要求。我们必须提升推动发展、管理社会、服务群众的能力和水平，毫不松懈地建设人民满意政府。

　　坚持依法行政。严格按照法定权限和程序行使职权、履行职责，规范行政行为。不断拓展政务公开的广度和深度，全市所有面向社会服务的政府部门要坚持办事公开制度，让权力在阳光下运行。加强机关效能建设，全面推行政府绩效管理。严格落实行政执法责任追究制，支持监察、审计部门依法履行监督职责，提高依法行政水平。

　　优化政务环境。深化行政审批制度改革，进一步清理、精简和调整全市行政审批和收费项目，坚持“两集中、两到位”，加快构建“审批一条龙、服务一站式”新机制。巩固提升市县乡村四级政务服务网络运管水平，积极推进市县联网审批，最大限度提高行政效能。深入开展“吃拿卡要”专项整治，严厉查处一切影响发展环境优化的违法违纪行为，强化对重要审批环节的监督检查，全力打造全省行政审批最少区、发展环境最优区。

　　狠抓工作落实。建立健全各项工作推进机制，落实“四化”率先发展领导组工作推进责任，继续实行县（区市）分组竞赛、经济运行分析调度、重点项目推进、工作督查常态化、安全生产例会等制度，深入推行一线工作法，确保各项工作落到实处。

　　强化廉洁自律。严格落实廉政建设责任制，坚持“一岗双责”，坚持反腐倡廉与经济工作一起部署、一起落实、一起考核。加强执法、司法、教育和廉政监察，运用“制度+科技”、“人控+技控”等手段，健全廉政风险防控机制，重点解决征地拆迁、住房保障、土地管理、食品药品安全、环境保护、安全生产等方面的突出问题。从严控制“三公”经费，继续实行零增长。

　　各位代表！我们所有的努力，就是要让广大人民群众的生活越来越好、更加幸福！

　　今年，市政府要在不折不扣落实省政府承诺为民办实事各项政策的基础上，挤出财力全力办好以下实事：一是开工建设城市保障性安居工程3.97万套，加强农村危房改造。二是新增城镇就业2.97万人，解决就业困难人员就业2459人。三是提高全市企业退休人员基本养老金，实现城乡居民养老保险全覆盖；分别提高城镇医疗保险和新农合标准40元，80岁以上城乡居民免费享受基本医疗保险；分别提高城乡低保标准30元、22元；分别提高农村集中供养、分散供养的五保对象补助标准500元、100元；为集中供养孤儿、散居孤儿每人每月分别补助1000元、600元。四是全面完成中等职业教育免学费全覆盖任务，新建和改扩建55所公办幼儿园。五是完成60条城市小街巷改造和129条城市街巷亮化任务，打造城市市民500米健身圈。六是全面完成农家书屋、乡镇邮政所建设全覆盖任务。

　　各位代表，扎实做好今年工作，加快建设全省“四化”率先发展区，是327万晋中人民的共同责任。让我们在中共晋中市委的坚强领导下，以强烈的责任感和使命感，励精图治，埋头苦干，锐意进取，全速推进转型跨越、率先发展步伐，以优异成绩迎接党的十八大胜利召开！
